# Study on Dielectric Function Models for Surface Plasmon Resonance Structure

**DOI:** 10.1155/2014/503749

**Published:** 2014-01-30

**Authors:** Peyman Jahanshahi, Mostafa Ghomeishi, Faisal Rafiq Mahamd Adikan

**Affiliations:** Photonics Research Group, Department of Electrical Engineering, Faculty of Engineering, University of Malaya, 50603 Kuala Lumpur, Malaysia

## Abstract

The most common permittivity function models are compared and identifying the best model for further studies is desired. For this study, simulations using several different models and an analytical analysis on a practical surface Plasmon structure were done with an accuracy of **∼**94.4% with respect to experimental data. Finite element method, combined with dielectric properties extracted from the Brendel-Bormann function model, was utilized, the latter being chosen from a comparative study on four available models.

## 1. Introduction

Surface plasmon resonance (SPR) has been exploited primarily in the area of sensing due to the level of sensitivity and selectivity that the technique offers. The ubiquity of the approach is evident in the amount of work undertaken and applications introduced over the years [1–5]. An excellent review work on the technology can be found in this reference [6, 7]. SPR takes advantage of the electromagnetic field behavior at a dielectric-metal interface, provided that the requisite phase matching conditions are fulfilled [8]. In most cases, the dielectric is glass whereas the metal can be either gold or silver [9, 10]. Practical SPR chips, however, require an additional layer to be introduced between these dielectric-metal interfaces to provide adhesion as gold/silver tends to peel away from the glass surface. Typical adhesive materials include titanium and chrome [11–13].

In most cases, SPR layers or chips display a very small fabrication tolerance. Errors in making the chips would highly influence important parameters such as the phase matching conditions, the wavelength to be utilized, and the launch angle. As such, it is common to first simulate the behavior of these chips and to estimate the level of tolerance that the completed chip is able to handle. In this respect, one of the most common approaches used is finite element analysis [14–17]. The technique allows denser calculation windows (triangulation) within the areas of interest in the multilayer SPR structure. However, the accuracy of the approach depends highly on the parameters used—in particular the dielectric and metal refractive indices and absorption coefficients at the wavelength of interest. Furthermore, the majority of the studies conducted so far have ignored the influence of the adhesive layer as it is believed that the nm thin layer poses no effect on the SPR behavior [15, 18–20].

The basic model in the field of optical properties of different materials is the well-known Drude model [21–23] and almost all the other models are improvements of this fundamental model. Subsequent to Drude, many scientists attempted to develop and refine new models, focusing mainly on experimentally nonparametric models [24, 25] that were not comprehensive or further developing the Drude model. The most applicable of these models are the Drude-Lorentz model [26–30], Brendel-Bormann model [26, 31], and multioscillator model [32–34] due to the fact that their results are more reliable than the other models; however, even these models are not well studied in different application conditions. Vial and Laroche [35] and Nunes et al. [36] described the dispersion properties of metals using Drude Model versus Drude-Lorentz model. In another work [37], a similar model discussion has been done by introducing two critical points in the Drude-Lorentz model. Comparison of these models looks interesting, but using verifiable experimental data as a reference for this kind of comparing appears necessary to prove the reliability of these models.

In this paper, we report an accurate simulation of an SPR structure that included the effect of adhesive layer. The dielectric and metallic properties of the layers involved were obtained by first comparing four function models and adopting the most accurate one—the Brendel-Bormaan (BB) function. We fed the parameters obtained from the BB model into our finite element analysis and showed that there is ~94.4% overlap with experimental data.

## 2. Dielectric Function Models

Dielectric function models are used to generate the refractive index and absorption coefficient values of dielectrics and metals at a particular wavelength, which will then be utilised to characterise dielectric-metal interface field interactions. In this work, we first compared four such models—Drude, Drude-Lorentz, Brendel-Bormann, and multiple oscillator. The comparison is necessary to assess the effect of some of the assumptions and simplifications made in generating these models towards the accuracy of the resulting values. In general, all the models are variants of the Drude model, with different levels of complexities.

### 2.1. Dielectric Function Models

In the most simplified cases of time domain methods for which the metal-dielectric frequency characterization is sought, the Drude model is invoked to approximate the dispersion properties of metals. In formula (1), *f*_0_ represents the oscillator strength (weight factor) and the plasma frequency *ω*_*p*_ is associated with intraband transitions (free-electron transitions) and is a function of electron density and mass which are given in Palik's Handbook of Optical Constants [38]. Scattering frequency Γ includes all the scattering interactions like electron-electron, electron-phonon, and so forth. However, a limited range of wavelength can be approximated by Drude model [21, 38]:

(1) $$\begin{array}{c} \varepsilon_{\text{Drude}}\left(\omega \right)=\varepsilon \left(\infty \right)-\;\frac{f_{0}\omega_{p}^{2}}{\omega \left(\omega -i\Gamma \right)}. \end{array}$$

Drude-Lorentz extends its range of validity by incorporating the separated interband (bound-electron) expression into the Drude model [35–37, 39]. The Drude-Lorentz model is an improvement of Drude model which takes into account the explicitly separated interband (bound-electron effects) expression to the initial model of Drude. By adding this Lorentzian term that is described by a semiquantum model, the validity range of Drude model can be extended [26]. In formula (2), *ω*_*j*_ and Γ_*j*_ are symbols of the oscillator resonant frequencies and bandwidths, respectively, *k* is the number of oscillators with frequency *ω*_*j*_, and *f*_*j*_ is oscillator strength or weight factor [26]. Despite the fact that the Drude-Lorentz model (D-L) expands the validity range of analytical metal-dielectric constants approximations, it is not appropriate for describing certain metal edges with sharp absorption. Several references stated that the approximation of the Drude-Lorentz model is not acceptable for noble metals (Cu, Ag, and Au) in the onset of interband absorption even for five Lorentzian terms [26]:

(2) $$\begin{array}{c} \varepsilon_{\text{D-L}}\left(\omega \right)=\varepsilon_{\text{Drude}}\left(\omega \right)+\overset{k}{\underset{j=1}{\sum }}\frac{f_{j}\omega_{j}^{2}}{\left(\omega_{j}^{2}-\omega^{2}\right)-i{\omega \Gamma }_{j}}. \end{array}$$

Brendel-Bormann reduces the errors generated from the Lorentzian function adopted in Drude by adopting a Gaussian complex error method instead [40]. This circumvents the erratic absorption bands displayed by Drude. In the broadening functions such as optical parameters in the Drude model, the Gaussian line shape functions give better results than Lorentzian line shape functions. If the same full width at half maximum (FWHM) and same weight are assumed for both of these functions, the Lorentzian function's wings are usually more extended and higher rather than the other function. As a result, all the Lorentzian based models show immoderate absorption in spite of what is expected [26]. The proposed model of Brendel and Bormann [31] for solids' dielectric function is based on a Gaussian complex error function (CEF) method [40] to reduce the deviance of Drude model from the real values. In this model the Lorentzian term in the Drude-Lorentz model is replaced by the Brendel-Bormann polynomial, *χ*_*j*_, as given by reference [26], which is improved by means of complex error function technique [40]. In the Brendel-Bormann (B-B) optical dielectric function (formula (3)), *k* is the number of B-B oscillators used to interpret the interband part of the spectrum [26, 31]. So a flexible shape for the absorption profile is obtainable via such an analytic function:

(3) $$\begin{array}{c} \varepsilon_{\text{B-B}}\left(\omega \right)=\varepsilon \left(\infty \right)-\frac{f_{0}\omega_{p}^{2}}{\omega \left(\omega -i\Gamma_{0}\right)}+\overset{k}{\underset{j=1}{\sum }}\chi_{j}\left(\omega \right). \end{array}$$

Multioscillator is also another interpretation of Lorentz model by adding different interband terms to extend the validity range of it which is used broadly among the scientists to calculate the wavelength dependent optical properties of metals [32]. Perhaps the most popular model for the metal-dielectric optical properties is the multiple oscillator model (M-O) which is based on the models of material oscillators. In the infrared and visible spectral ranges, the wavelength dependencies in optical indices for thin metal films are very complicated and one of the models that had a very good response in this case was the multiple oscillator model [32, 34, 41]. In this formula (4) *ω*_*pj*_ is the plasmonic resonant frequency of the oscillators and Γ_*j*_ is its related bandwidths. Again *f*_*j*_ is a weight factor and *k* is the oscillators counter:

(4) εM-O(ω)=ε(∞)−ωf0ωp2−if0ωp2Γω(ω2+Γ02) +∑j=1kfjωpj2(−ω2+ωpj2)+iωfjΓjωpj2ω2Γj2+(−ω2+ωpj2)2.

The experimental data has been chosen carefully to provide us an acceptable reference for the optical models used in our study [38, 42–46]. The absorption coefficients of some metals including gold which has been published by Schulz [43] are used in this paper. For the refractive indices another database which was a collaboration of Schulz and Tangherlini [42], using the same metals was utilized.

## 3. Results and Discussions

### 3.1. Simulation with Different Dielectric Function Models

**Figure 1:**
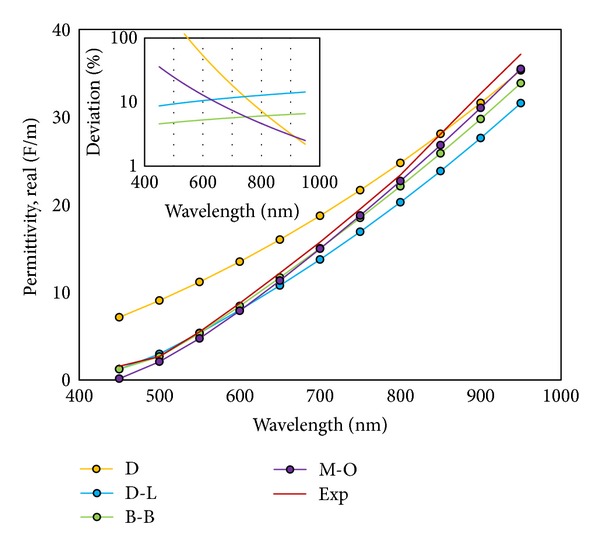
Real part of permittivity (F/m) against wavelength (nm) for all four models compared to experimental data. Inset: error value of the permittivity against wavelength indicating the accuracy of the B-B model with less than 10% deviation.

**Figure 2:**
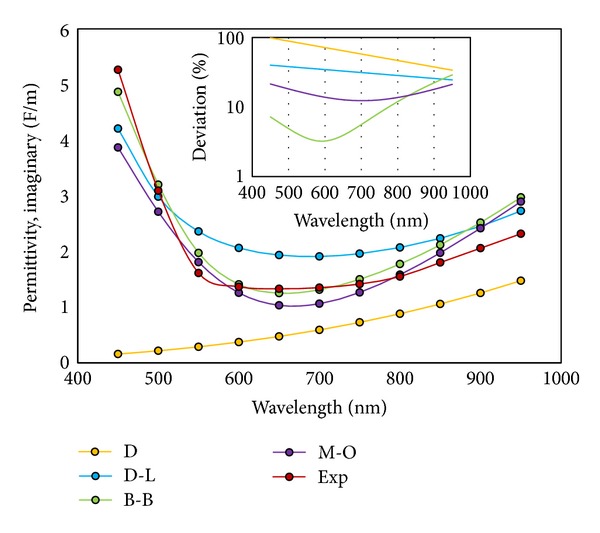
Imaginary part of permittivity (F/m) against wavelength (nm) for all four models compared to experimental data. Inset: error value of the permittivity against wavelength indicating the accuracy of the B-B model with less than 10% deviation.

We performed simulations using the above four models and compared the results with experimental data extracted from references. The measured optical constants in those datasheets have been done at the wavelength range of 450 nm to 950 nm at glass/air-metal interfaces. Figures 1 and 2 depict the results of the simulation and the error analysis with respect to experimental data.

### 3.2. Analytical and Numerical Analysis of the Models on SPR Structure

**Figure 3:**
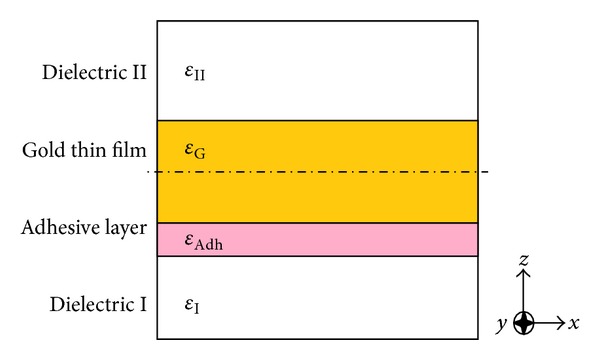
Practical waveguide structure (SPR), from up to down: dielectric II, gold, titanium and then dielectric I.

As evident from Figures 1 and 2, some function models are superior to others. The Brendel-Bormann and the multioscillator models have more overlap with the experimental data in both real and imaginary parts of the permittivity and it can be determined via deviation curves. As a final check, we implemented all four models in simulating a structure depicted in Figure 3. The multilayer structure shown utilized the specific optical properties generated by the various models. The results in the form of percentage of reflected power and plasmonic reflectance angle were of interest in identifying the most accurate of the four models. In the diagram, dielectric I is silica with infinite thickness, the adhesive layer is 3 nm titanium, a 50 nm gold thin film, and BK7 acts as dielectric II with infinite thickness.

**Figure 4:**
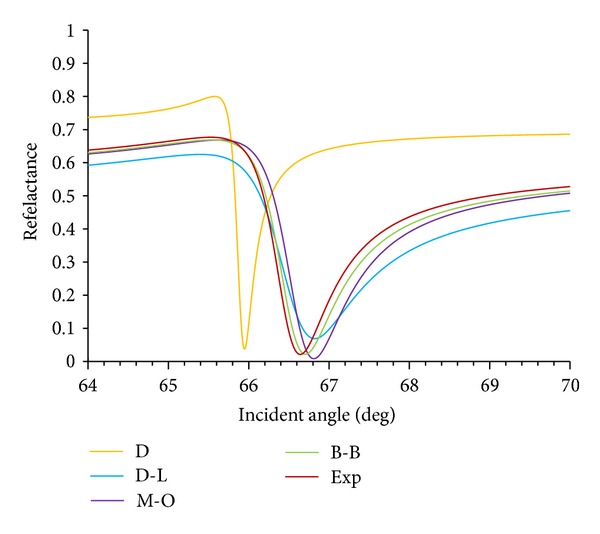
Reflectance versus incident angle of 30° to 70°, at 600 nm.

In theory, the SPR angle is an incident angle where the entire beam is absorbed by the layers to make a maximum resonance and as a result there will be no reflection from the structure. The optical properties of different layers in the SPR structure can affect this special angle. On the other hand, any perturbation on the surface of this structure can shift the SPR angle which is the basis of its sensing ability. It is very important to estimate the most accurate SPR angle, shown in Figure 4, using different models. The dips in this plot indicate the lowest reflectance in the SPR angle. Each continuum belongs to an optical model that was applied in calculating the permittivity of metallic layers. Based on our experimental observation, the plasmon resonances on gold surface of the proposed SPR structure occurred at the operating wavelength of 600 nm which is utilized in this study. It appears that the B-B model has the best fit with the experimental continuum followed by the M-O model. The experimental fit was obtained from the practical permittivity data published by Schulz and his team [42, 43].

**Figure 5:**
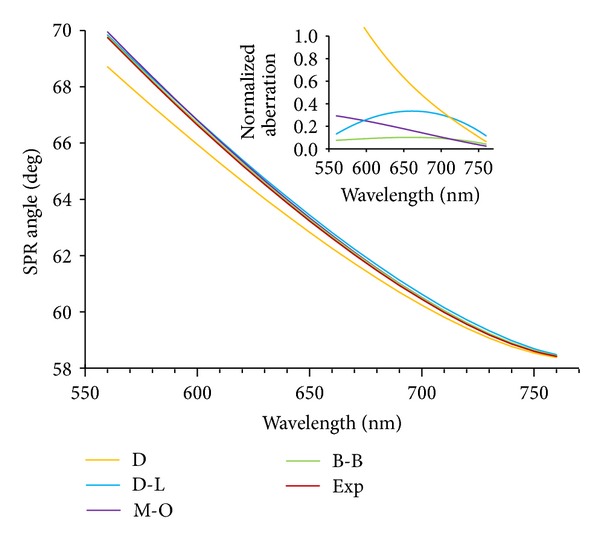
(a) SPR angle (degree) versus wavelength of 560 nm to 760 nm. Inset: error value at SPR angle for the same wavelength.

After finding the SPR angle for a specific wavelength, we tried to calculate it for different wavelengths using dielectric function models. In Figure 5 the SPR angle is calculated with respect to the permittivity of gold and titanium films which directly depends on wavelength. For each wavelength, the optical properties of the thin films cause the change in SPR angle. Scientists should take attention to the fact that even a very small aberration in the angle value can affect the tuning of the SPR setup.

**Figure 6:**
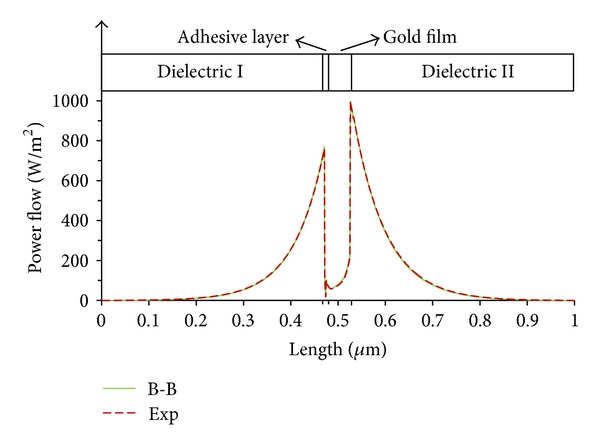
Comparison between using experimental data and Brendel-Bormann model at energy density time average distribution of the light.

Finally, we ran a numerical simulation of the SPR structure via finite element analysis. The FEA model employs dielectric and metallic properties obtained experimentally and those generated by the B-B model.  Figure 6 shows the power flow of the SPR structure which is normalized by time average. The gradual increase in the power flow collapses suddenly at the dielectric-metal interface and vice versa, while a small sharp dip can be detected at the titanium and gold boundary. This implies that the surface sensing increases when there is a dielectric-metal boundary compared to the metal-metal boundary. The field is coupled into a plasmon mode, which propagates at the boundary, forming a surface mode. In our case, it is clear that the increase of the continuum is sharp, and the sensitivity is very high near the gold wall. The average aberration of B-B model against the experimental data is about 2.2 W/m^2^ which means the B-B continuum has 94.4% overlap with the experimental continuum.

## 4. Conclusion

In this work we applied Drude, Drude-Lorentz, Brendel-Bormann, and Multiple Oscillator models to determine which model is more compatible and reliable for SPR sensing studies. The Brendel-Bormann model was found to be the most accurate in generating real and imaginary permittivity values for the materials used in the proposed SPR structure, within the wavelengths of interest. We have also considered the influence of the thin titanium layer used to fix the gold thin film on a glass substrate in both analytical and numerical analyses.
